# Decoding the Gut Microbiome in Primary Sjögren’s Syndrome and Primary Biliary Cholangitis: Shared Dysbiosis, Distinct Patterns, and Associations with Clinical Features

**DOI:** 10.3390/microorganisms13122668

**Published:** 2025-11-24

**Authors:** Bo Zang, Lishan Xu, Haojie Huang, Qixuan Liu, Yuan Yao, Jiaxiu Li, Yifei Yang, Chenyang Zhao, Bingqian Liu, Bin Liu

**Affiliations:** 1 Department of Rheumatology, Affiliated Hospital of Qingdao University, Qingdao 266001, China; zangbo95@163.com (B.Z.); jiaxiuli3778@163.com (J.L.); yfeiyang2021@163.com (Y.Y.);; 2 Department of Rheumatology, Liaocheng People’s Hospital, Liaocheng 252003, China; 3 State Key Laboratory of Microbial Technology, Shandong University, Qingdao 266299, China; 4 Graduate Group of Epidemiology, School of Veterinary Medicine, University of California, Davis, CA 95616, USA; 5 Department of Internal Medicine II, Heart Center Bonn, University Hospital Bonn, 53127 Bonn, Germany

**Keywords:** gut microbiome, primary biliary cholangitis, primary Sjögren’s syndrome, 16S rRNA gene sequencing, clinical indicators, key microbial marker

## Abstract

This study aimed to analyze gut microbiome similarities and differences between primary biliary cholangitis (PBC) and primary Sjögren’s syndrome (pSS), exploring potential associations with disease pathogenesis. High-throughput sequencing of the 16S rRNA gene was performed on fecal samples from 100 subjects (PBC: 38; pSS: 42; HC: 20) to compare the composition, diversity, and key microbial markers, examining associations with clinical indicators. The gut microbiome of PBC and pSS patients exhibited reduced alpha diversity (*p* < 0.05) and decreased abundance of the Bacteroides genus (both *p* < 0.001). While the majority of differentially abundant species were similar in PBC and pSS, unique imbalances were noted: Actinobacteria was elevated in pSS, whereas Proteobacteria was higher in PBC (*p* < 0.05). At the species level, a higher relative abundance of *Ruminococcus torques*, *Clostridium celatum*, and *Lactobacillus vaginalis* was identified in PBC patients, with positive correlations observed with key clinical indicators such as liver enzymes and TBA. In pSS patients, *Faecalibacterium prausnitzii* showed a negative correlation with GGT and ALT. Although PBC and pSS shared many similarities in their gut microbiome’s composition and diversity, indicating common mechanistic microbial influences on their pathogenicity, distinct microbial profiles correlated with clinical indicators in each disease, highlighting specific microbiome–disease interactions that may underlie their differential pathogenesis.

## 1. Introduction

Primary biliary cholangitis (PBC) and primary Sjögren’s syndrome (pSS) are both immunological disorders, histopathologically characterized as autoimmune epithelitis, involving immune-mediated epithelial cell damage [1]. Whether they should be classified as distinct or related disorders remains subject to debate. PBC involves the targeted destruction of biliary epithelial cells, leading to non-suppurative damage to the intrahepatic bile ducts [2], whereas pSS primarily targets exocrine glands, though it can also affect the liver. More than 20% of pSS patients exhibit liver function abnormalities and hepatic pathologies that resemble those seen in PBC [3,4]. The co-occurrence of pSS and PBC is relatively common, suggesting a possible overlap in pathophysiology. These associations imply that PBC and pSS may share common pathogenic factors and mechanisms of disease development.

An increasing number of studies have suggested a role for gut dysbiosis in the pathogenesis and progression of both PBC and pSS [5,6]. Patients with either disease often exhibit reduced gut microbial diversity, a marker of microbiome stability [7,8]. While previous studies have found notable differences in gut microbiome composition between PBC and pSS patients and healthy controls (HCs), the specific profiles and their clinical correlations require further elucidation. Given the observed clinical and pathological overlaps between PBC and pSS, characterizing the similarities and differences in gut microbiome composition between these diseases is crucial. An altered gut microbiome may impact disease activity and progression via the gut–liver and gut–immune axes, potentially contributing to pathogenesis through pro-inflammatory pathways and immune dysregulation, thereby warranting deeper investigation.

This study aims to analyze the similarities and differences in gut microbiome composition between PBC and pSS patients and to identify microbial markers linked with clinical indicators of disease severity. Our findings aim to enhance the understanding of microbiome–disease interactions that may underpin the pathogenesis and progression of PBC and pSS.

## 2. Materials and Methods

### 2.1. Study Group

Patients with PBC and pSS were recruited from the Department of Rheumatology and Immunology at an affiliated hospital of Qingdao University, China, between December 2019 and June 2020. Thirty-eight PBC patients were diagnosed according to the 2000 American Association for the Study of Liver Diseases (AASLD) PBC guidelines [9]. Based on serum alkaline phosphatase (ALP) levels, PBC patients were divided into mild and severe groups: the mild group included patients with ALP levels ≤ 3 times the upper limit of normal (ULN), while the severe group comprised patients with ALP levels > 3 times the ULN. Forty-two pSS patients were enrolled according to the 2012 American College of Rheumatology (ACR) Classification Criteria for Sjögren’s Syndrome [10]. pSS patients with EULAR Sjögren’s Syndrome Disease Activity Index (ESSDAI) scores > 3 were assigned to the active group, while those with scores ≤ 3 were placed in the inactive group. Additionally, 20 healthy subjects were recruited as controls from among individuals undergoing routine health examinations at the hospital’s Health Management Center. All HCs met the following inclusion criteria: (1) normal blood pressure; (2) normal results for urine, stool, blood glucose, lipid, and kidney function tests; (3) negative for hepatitis B/C virus antigens; and (4) no antibiotic, prebiotic, or probiotic use within the previous three months. Potential confounding variables, including diet and medication use, were carefully considered and recorded; analysis confirmed no significant differences in the intake of key dietary components (e.g., total dietary fiber and alcohol) among the groups (*p* > 0.05). This study was approved by the Ethics Committee of the Affiliated Hospital of Qingdao University (Approval No. QYFY WZLL 26798), and written informed consent was obtained from all participants for sample collection and study participation.

### 2.2. DNA Extraction, 16S rRNA Gene Amplicon Sequencing and Sequence Analysis

Fecal samples were collected, frozen at −80 °C within two hours, and processed for genomic DNA extraction using the OMEGA Soil DNA Kit (Omega Bio-Tek, Norcross, GA, USA). DNA quality and concentration were assessed via a NanoDrop NC2000 spectrophotometer (Thermo Fisher Scientific, Waltham, MA, USA) and agarose gel electrophoresis.

The V3–V4 region of the 16S rRNA gene was amplified using the primers 338F (5′-ACTCCTACGGGAGGCAGCA-3′) and 806R (5′-GGACTACHVGGGTWTCTAAT-3′) with unique barcodes. The PCR mixture included a buffer, Fast pfu DNA polymerase, dNTPs, primers, template DNA, and ddH_2_O. The thermal cycling conditions were as follows: initial denaturation at 98 °C for 5 min, 25 cycles of denaturation at 98 °C for 30 s, annealing at 53 °C for 30 s, and extension at 72 °C for 45 s, followed by a final 5 min extension at 72 °C. PCR products were purified using VAHTS™ DNA Clean Beads (Vazyme, Nanjing, China) and quantified with the Quant-iT PicoGreen dsDNA Assay Kit (Invitrogen, Carlsbad, CA, USA). Amplicons were pooled equally and sequenced using the Illumina NovaSeq platform (Shanghai Personal Biotechnology Co., Ltd., Shanghai, China).

Sequence data were analyzed with QIIME2 [11]. Demultiplexing and primer trimming were performed with the demux and cutadapt plugins, while DADA2 was used for quality control, denoising, merging, and chimera removal. ASVs were aligned with MAFFT, and a phylogenetic tree was constructed using FastTree2. Alpha (Shannon index) and beta diversity (weighted UniFrac) metrics were calculated, and taxonomic classification was performed with the classify-sklearn tool using the SILVA 132 database.

### 2.3. Bioinformatics and Statistical Analysis

Analysis of sequence data was primarily conducted using the QIIME2 and R packages (v3.2.0). Shannon diversity indices based on ASVs were calculated in QIIME2 and displayed as box plots. Beta diversity was examined to assess microbial community structure variation using UniFrac distance metrics [12] and visualized via principal coordinate analysis (PCoA) and hierarchical clustering [13]. Taxonomic composition and relative abundances were displayed using MEGAN (GenesCloud, https://www.genescloud.cn) [14] and GraPhlAn (GenesCloud, https://www.genescloud.cn) [15]. Shared and unique ASVs among groups were illustrated with Venn diagrams generated using the “VennDiagram” R package (GenesCloud, https://www.genescloud.cn), based on ASV presence across samples or groups, irrespective of relative abundance [16].

### 2.4. Statistical Analyses

Continuous variables are expressed as the mean ± the standard deviation (SD) or the interquartile range and were analyzed using either ANOVA or the Mann–Whitney U test for comparisons across groups. Categorical data were compared among groups using the chi-square test. LEfSe analysis was conducted with the R microeco package. Correlations were examined through Spearman correlation analysis. A *p*-value of less than 0.05 was deemed statistically significant. Data analyses were carried out using SPSS version 25.0 and R version 3.6.0.

## 3. Results

### 3.1. Clinical Indicators of PBC, pSS, and HCs

The demographics and clinical characteristics of participants are summarized in Table 1. After quality control, a total of 100 fecal samples were obtained from PBC and pSS patients and HC subjects. There were no significant differences in age, gender, height, weight, and body mass index (BMI) among all participants.

The comparison of serological indexes across groups (Table 2) revealed that alanine aminotransferase (ALT), aspartate aminotransferase (AST), gamma-glutamyl transpeptidase (GGT), ALP, total bile acid (TBA), and immunoglobulin M (IgM) levels in PBC patients were significantly higher than those in the HC group (all *p* < 0.05). These indicators, except for IgM, were also significantly elevated in PBC patients compared to the pSS group. The immunoglobulin G (IgG) level was significantly higher in pSS patients than in both the HC and PBC groups (*p* < 0.001 and *p* = 0.013, respectively). Additionally, erythrocyte sedimentation rate (ESR) levels were significantly elevated in both the pSS and PBC groups compared to HCs (both *p* < 0.001). Among the complement components, complement component 3 (C3) was slightly lower in pSS patients than in HCs, while it was higher in PBC patients, though these differences were not significant. There were no significant differences in immunoglobulin A (IgA) and complement component 4 (C4) levels across the three groups.

Comparisons of clinical indicators in pSS and PBC patients under different disease stratifications are presented in Table 3 and Table 4, respectively. Active pSS patients exhibited significantly lower platelet (PLT) levels compared to inactive patients (*p* < 0.001), along with lower C3 levels (*p* = 0.002). Additionally, IgG levels were markedly higher in active pSS patients than in inactive patients (*p* < 0.001). Severe cholestasis in PBC patients was associated with significantly higher levels of ALT, AST, GGT, ALP, and TBA compared to mild cholestasis (all *p*-values < 0.05).

### 3.2. Differences in Gut Microbiome Composition

**Figure 1:**
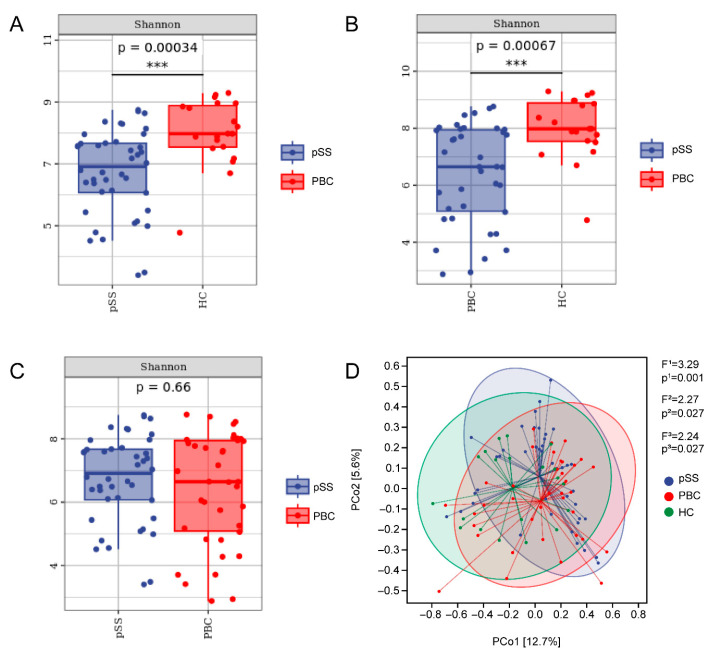
Gut microbiota diversity in patients with PBC and pSS and HCs. (**A**–**C**) Comparison of alpha diversity among the PBC, pSS, and HC groups using the Shannon diversity index, which integrates species richness and evenness. (**D**) Principal coordinate analysis (PCoA) plot based on weighted UniFrac distances, illustrating the clustering of the gut microbiota composition across the three groups. *** *p* < 0.001.

A total of 64,141 sequenced ASVs were processed from all of the fecal samples. The number of sequenced ASVs obtained from PBC, pSS, and HC samples was 18,243, 19,913 and 25,985, respectively. Both the pSS and PBC cohort had a reduced gut microbiome α-diversity, as measured by abundance, compared with the HCs (Shannon, *p* = 0.00034, Figure 1A; *p* = 0.00067, Figure 1B), while there were no differences in the evenness and richness of the gut microbiome between the PBC and pSS patients (Shannon, *p* = 0.66, Figure 1C). A weighted UniFrac principal coordinate analysis (PCoA) was performed to identify the differences in gut microbiome composition between patients with PBC and pSS and HCs. As shown in Figure 1D, there were significant differences in the clustering for PBC and pSS patients compared with HCs (*p*^1^ = 0.001, *p*^2^ = 0.027 and *p*^3^ = 0.027).

**Figure 2:**
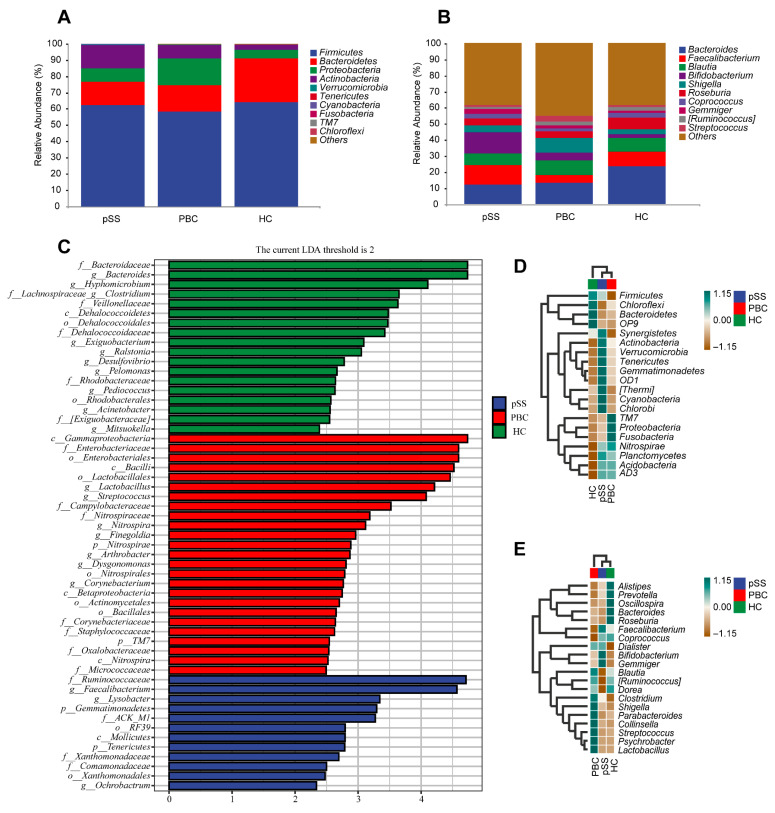
Composition of relative abundance of gut microbiota at the phylum/genus level in PBC, pSS patients, and HCs. (**A**) Comparison of the relative abundance of the top 10 gut microbiota at the phylum level in pSS and PBC patients and HCs. (**B**) Comparison of the relative abundance of the top 10 gut microbiota at the genus level in pSS and PBC patients and HCs. (**C**) Linear discriminant analysis (LDA) demonstrated distinct bacterial genera enriched in pSS and PBC patients and HCs. Genera with *p* < 0.05 and an LDA score > 2 were considered significant and are shown here with notations for their corresponding phylum (*p*), family (f), order (o), class (c), and genus (g) level. (**D**) Variations in the heatmap of the top 20 abundances of fecal bacterial taxa at the phylum level. (**E**) Variations in the heatmap of the top 20 abundances of fecal bacterial taxa at the genus level.

According to the annotation, the gut microbiome of the PBC, pSS, and HC groups consisted of four bacterial phyla, specifically Firmicutes, Bacteroidetes, Proteobacteria, and Actinobacteria. Firmicutes (58.45%, 62.42% vs. 64.21%; *p* = 0.145, 0.639) and Bacteroides (14.25%, 16.17% vs. 26.77%; *p* = 0.378, 0.062) levels in the PBC and pSS patients were lower than in the HCs. The Proteobacteria levels in PBC patients were higher than in pSS patients and HCs (16.36% vs. 8.15%, 5.51%; *p* = 0.025, 0.001). The Actinobacteria levels in PBC and pSS patients were higher compared with HCs (8.45%, 14.31% vs. 3.16%; *p* = 0.039, 0.000) (Table S1, Figure 2A). These results indicate that the composition of the gut microbiome was different in the two groups for Actinobacteria and Proteobacteria when compared with HCs. At the genus level, analysis of the top 10 most abundant gut microbiomes revealed a significant reduction in Bacteroides abundance in both pSS and PBC patients compared with HCs, with a more pronounced reduction in the pSS group (11.74%, 13.04% vs. 23.32%, both *p* < 0.001). The proportion of Faecalibacterium in the pSS group was significantly higher than in the PBC group (12.34% vs. 5.01%, *p* = 0.004). Interestingly, Bifidobacterium was more abundant in pSS patients compared to HCs and PBC patients (13.13% vs. 2.55%, 5.08%; *p* < 0.001, *p* = 0.001) (Table S2, Figure 2B). To determine the differences in the gut microbiota among the three groups, the abundances at the genus level were compared using linear discriminant analysis effect size (LEfSe) (Figure 2C). Twenty-five biomarkers were found in PBC patients, with the top five being c__Gammaproteobacteria, f__Enterobacteriaceae, o__Enterobacteriales, c__Bacilli, and o__Lactobacillales, with c__Gammaproteobacteria being the most significant. In pSS patients, the main biomarkers were f__Ruminococcaceae, g__Faecalibacterium, g__Lysobacter, *p*__Gemmatimonadetes, and f__ACK_M1. In the HC group, the primary biomarkers included f__Bacteroidaceae, g__Bacteroides, g__Hyphomicrobium, f__Lachnospiraceae_g__Clostridium, and f__Veillonellaceae. The similarities and differences in gut microbiome composition among the pSS, PBC, and HC groups were further explored using heat map analysis, which revealed significant variations in microbial abundance across the groups. At the phylum level (Figure 2D), the relative abundance of Saccharibacteria (TM7), Proteobacteria, and Fusobacteria was notably higher in the PBC group. In the pSS group, Synergistes, Actinobacteria, Verrucomicrobia, Tenericutes, and Gemmatimonadetes showed increased abundance. Conversely, the HC group exhibited higher relative abundance of Firmicutes, Bacteroidetes, Chloroflexi, and Caldatribacterium (OP9). The relative abundance of the top 20 gut microbes at the genus level (Figure 2E) revealed that Lactobacillus, Psychrobacter, Streptococcus, Collinsella, Parabacteroides, and Shigella were dominant in PBC patients, whereas Bifidobacterium, Gemmiger, and Faecalibacterium were dominant in pSS patients. In HCs, Bacteroides, Oscillospira, Roseburia, Alistipes, and Prevotella were the most dominant. Based on this data, the abundance of pathogenic bacteria (Collinsella, Shigella) in the gut microbiome in patients with PBC was higher than in pSS patients and HCs.

### 3.3. Serological Chemistry and Gut Microbiome Comparison Based on Disease Severity in PBC and pSS Patients

**Figure 3:**
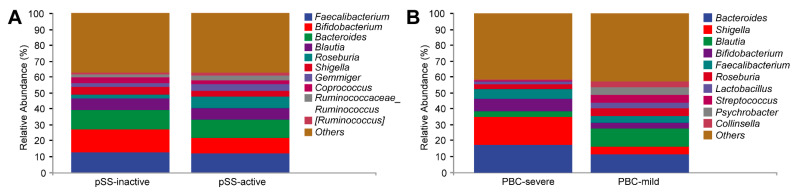
Relative abundance of gut microbiota based on disease severity in PBC and pSS patients. (**A**) Relative abundance of the top 10 genera in the fecal microbiome of active and inactive pSS patients. (**B**) Relative abundance of the top 10 genera in the fecal microbiome of patients with mild and severe cholestasis.

At the genus level, the relative abundance of the top 10 most prevalent bacterial genera varied across different levels of disease activity. In the active disease group, the proportion of Roseburia was significantly higher (*p* = 0.002), while the proportion of Bifidobacterium was lower, although this difference did not reach statistical significance (Table S3, Figure 3A).

The relative abundance of Blautia was markedly lower in the severe cholestasis group compared to the mild cholestasis group (*p* = 0.003). While Shigella showed higher abundance and Corprococcus displayed lower abundance in the severe cholestasis group, these differences did not reach statistical significance (Table S4, Figure 3B).

### 3.4. Characteristic Species of the PBC, pSS, and HC Groups and Their Correlation with Clinical Indicators

**Figure 4:**
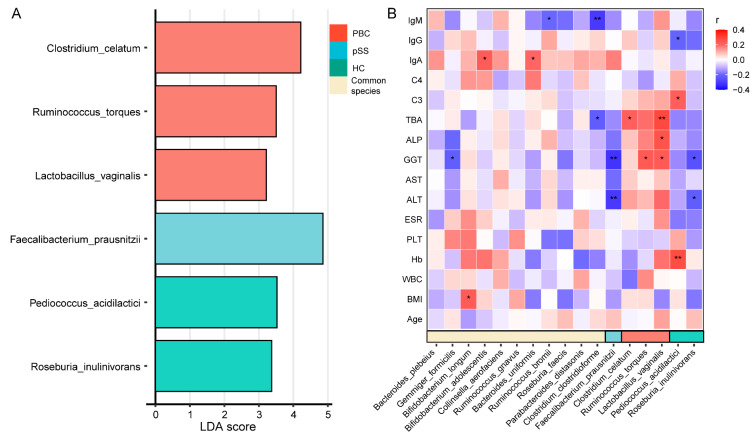
Characteristic species of the PBC, pSS, and HC groups and correlation with clinical indices. (**A**) Linear discriminant analysis (LDA) of gut microbial species identified characteristic genera for the PBC (red), pSS (blue), and HC (green) groups, along with common species shared across groups (yellow). The LDA score indicates the relative importance of each species in distinguishing among groups. (**B**) Heatmap of correlations between gut microbial species and clinical indices. Red represents a positive correlation, blue represents a negative correlation, and white represents no correlation. * *p* < 0.05; ** *p* < 0.01.

A high-dimensional biomarker and linear discriminant analysis was conducted, using LEfSe, on the gut microbiome of study subjects to identify key microbial markers. As shown in Figure 4, an LDA score threshold of two was set to distinguish the species specific to each group. *Clostridium celatum*, *Ruminococcus torques*, and *Lactobacillus vaginalis* were identified as characteristic of the PBC group, *Faecalibacterium prausnitzii* was identified as specific to the pSS group, and *Pediococcus acidilactici* and *Roseburia inulinivorans* were observed to be abundant in the HC group.

Spearman correlation analysis was performed to investigate associations between these characteristic species and clinical indicators. The results suggest that, in PBC patients, *Clostridium celatum* was found to be positively associated with TBA, *Ruminococcus torques* was found to be positively associated with GGT, and *Lactobacillus vaginalis* was found to be positively associated with TBA, ALP, and GGT. In pSS patients, *Faecalibacterium prausnitzii* was negatively associated with GGT and ALT, while in the HC group, *Pediococcus acidilactici* was positively correlated with C3 and hemoglobin (Hb) and negatively correlated with IgG, and *Roseburia inulinivorans* was negatively associated with GGT and ALT. Additional analyses indicated that *Gemmiger formicilis* was negatively correlated with GGT, *Bifidobacterium longum* was positively correlated with BMI, *Bifidobacterium adolescentis* and *Bacteroides uniformis* were positively correlated with IgA, *Ruminococcus bromii* was negatively correlated with IgM, and *Clostridium clostridioforme* was negatively correlated with TBA and IgM.

## 4. Discussion

Alterations in the gut microbiota composition are considered potential pathogenic mechanisms in PBC and pSS [6,8]. Our data reveal several shared microbial changes in the gut microbiomes of PBC and pSS patients, suggesting common microbial influences. However, disease-specific associations with clinical indicators highlight distinct pathological features.

Reduced gut microbiome alpha diversity was observed in both PBC and pSS patients compared to HCs, with no significant difference between the two diseases. Lower diversity might promote pathogenic bacteria niches, disrupt the mucosal barrier, and trigger inflammatory responses, leading to autoimmune diseases (AIDs) [17]. In sterile environments, pSS mice exhibited earlier and more severe mucosal damage, inversely related to fecal flora diversity [18,19]. These findings suggest that diminished gut microbiome diversity contributes to PBC and pSS pathogenesis. At the phylum level, an increased abundance of *Proteobacteria* was observed in PBC patients, which included many AID-causing pathogens. Animal studies confirm that elevated *Proteobacteria* levels disrupt gut microbiome stability and serve as biomarkers of dysbiosis and disease [20]. These bacteria produce endotoxins, activate dendritic cells, and promote pro-inflammatory mediators, leading to lymphoid hyperactivation and AIDs. Thus, *Proteobacteria* likely play a role in PBC onset. At the genus level, the relative abundance of *Bacteroides* was significantly reduced in PBC and pSS patients compared to HCs. *Bacteroides* plays a critical role in maintaining gut homeostasis, dietary fiber degradation, and bile acid metabolism [21,22]. Its metabolites, particularly short-chain fatty acids, strengthen the intestinal barrier and suppress inflammatory responses. Studies have linked reduced *Bacteroides* abundance to intestinal barrier disruption and immune dysregulation [23], which may exacerbate the pathogenesis of PBC and pSS. Restoring *Bacteroides* abundance or its metabolic functions may therefore represent a promising therapeutic strategy. Furthermore, an increased abundance of *Collinsella* and *Shigella* was identified in PBC patients, particularly those with severe cholestasis, similar to patterns in rheumatoid arthritis and Graves’ disease [24,25]. Metabolites from these bacteria reduce tight junction protein ZO-1, increase intestinal permeability, and upregulate RORα and chemokines, enabling pathogenic invasion and triggering immune responses [26]. *Shigella* further invades the colonic epithelium through the virulence factors IpaJ and VirA [27]. These bacteria and their metabolites may enter the liver via the portal system, exacerbating inflammation and liver damage. It is speculated that *Collinsella* and *Shigella* contribute to the pathogenesis of PBC.

Additionally, patterns of gut microbiota dysbiosis have been associated with disease progression. Studies have shown that *Blautia* plays a role in immune regulation [28]. In the present study, the proportion of *Blautia* was significantly lower in the PBC group with severe cholestasis compared to the mild cholestasis group. Studies have shown that *Roseburia* plays a role in reducing inflammation and enhancing intestinal barrier function [29]. However, in this study, *Roseburia* levels were significantly increased in patients with active pSS. These findings suggest that the balance within the gut microbiota undergoes dynamic changes with disease progression. However, the underlying mechanisms remain unclear and warrant further investigation.

To further delineate the alterations in the gut microbiome between PBC and pSS and their potential impact on the host, we identified key microbial markers at the species level and examined their correlations with clinical parameters. PBC patients have higher relative abundances of *R. torques*, *C. celatum*, and *L. vaginalis*, all of which positively correlate with key clinical indicators such as liver enzymes and TBA, highlighting the role of specific gut dysbiosis in PBC pathogenesis. *R. torques* showed a positive correlation with GGT levels in our study. It converts bile acids to deoxycholic acid (DCA), modulating immune responses through the FXR and TGR5 pathways, typically with anti-inflammatory effects [30,31]. However, DCA may act as a pro-inflammatory FXR antagonist in the presence of chenodeoxycholic acid [32]. Additionally, *R. torques* degrades intestinal mucin, potentially disrupting gut barriers and enabling microbial translocation to the portal vein [33]. This dual role warrants further investigation into its pathogenic or therapeutic implications in PBC. Moreover, the association of *C. celatum* with TBA suggests its role in bile acid metabolism. Bile acids act as signaling molecules, influencing immunity and microbiota balance, consistent with Thangamani et al. [34]. Abnormal bile acid metabolism may worsen liver injury and inflammation in PBC through TLR pathways, indicating that *C. celatum* may contribute to bile acid dysregulation and immune modulation in PBC. The positive correlations of *L. vaginalis* with TBA and various liver enzymes suggest a potential role in PBC progression. Increased *Lactobacillus* abundance was observed in PBC patients [35], and in a case where PBC developed following the administration of a *Lactobacillus* vaccine for recurrent vaginitis, experimental evidence indicated that *Lactobacillus* mimics the E2 subunit, triggering anti-mitochondrial antibody responses, suggesting that *Lactobacillus* vaccines may be another cause of PBC in genetically susceptible women [36]. Limited research suggests that elevated *L. vaginalis* abundance in the gut can provoke immune responses [37], warranting further investigation. In pSS patients, *F. prausnitzii* negatively correlates with GGT and ALT, suggesting its potential to alleviate hepatic inflammation. Through butyrate production, *F. prausnitzii* supports intestinal integrity and exerts anti-inflammatory effects [38], consistent with its known protective role in inflammatory liver injury [39]. In the HC group, *P. acidilactici* positively correlates with C3 and Hb and negatively correlates with IgG, reflecting its probiotic role in anti-inflammatory and immune regulation by increasing complement levels and balancing immunoglobulin responses, which is consistent with the findings of Maniat, M. et al. [40]. Additionally, *R. inulinivorans* is negatively correlated with GGT and ALT in the HC group. Studies indicate that *R. inulinivorans* produces butyrate, exerting anti-inflammatory effects on the liver and gut and potentially lowering liver enzyme levels, consistent with our results [41,42].

This study also has various limitations, such as the fact that the immunologic and lymphocyte subsets and cytokine levels were not assessed between the three groups. The association between inflammation and microbiome alterations was not studied in detail. An additional limitation is the use of the 2012 ACR criteria rather than the updated 2016 ACR/EULAR classification criteria for diagnosing pSS patients. Although these two sets of criteria demonstrate a high degree of overlap in identifying definite pSS cases, future studies utilizing the latest criteria for validation would be highly beneficial.

## 5. Conclusions

In conclusion, the gut microbiomes of patients with PBC and pSS showed significant increases in pro-inflammatory bacteria and opportunistic pathogens and a reduction in beneficial bacteria. PBC and pSS patients exhibited a number of similar changes in their gut microbiome, suggesting common microbial influences. However, disease-specific associations with clinical indicators reveal unique pathological profiles. In PBC, higher abundances of *Ruminococcus torques*, *Clostridium celatum,* and *Lactobacillus vaginalis* correlate positively with liver enzymes and TBA, while *Faecalibacterium prausnitzii* in pSS shows negative correlations with GGT and ALT. These disease-specific microbial associations underscore the distinct roles of the gut microbiota in the pathogenesis of PBC and pSS, offering a foundation for targeted microbiome-based therapeutic strategies. This study provides valuable insights into the role of gut microbes in diseases with similar pathophysiologies, as well as potential key microbial markers for screening and diagnostic validation in PBC and pSS patients.

## Figures and Tables

**Table 1:** Characteristics of PBC and pSS patients and HCs.

		PBC *n* = 38	pSS *n* = 42	HC *n* = 20	*p*-Value
Age in years, mean ± SD		46.24 ± 7.74	45.43 ± 10.81	44.85 ± 8.22	0.85
Gender, *n*	Male	2	3	1	1.00
	Female	36	39	19	
Height (cm)		160.45 ± 4.49	161.29 ± 3.78	160.70 ± 5.30	0.69
Weight (kg)		57.68 ± 4.66	57.57 ± 4.93	56.40 ± 5.63	0.61
BMI (kg/m^2^)		22.45 ± 2.10	22.12 ± 1.53	21.82 ± 1.61	0.42

PBC: primary biliary cholangitis; pSS: primary Sjögren’s syndrome; HC: healthy controls; BMI: body mass index.

**Table 2:** Comparison of serological indexes between PBC and pSS patients and HCs.

	pSS	PBC	HC	*p* ^a^-Value	*p* ^b^-Value	*p* ^c^-Value
WBC (10~9/L)	4.86 ± 1.62	6.19 ± 2.16	5.19 ± 1.21	0.420	0.062	0.002 **
Hb (g/L)	130 ± 12	126 ± 14	128 ± 13	0.679	0.512	0.188
PLT (10~9/L)	195 (162, 226)	153 (128, 242)	160 (148, 187)	0.012 *	0.623	0.114
ESR (mm/1 h)	18 (12, 27)	14 (11, 23)	5.55 (4.15, 8.15)	<0.001 ***	<0.001 ***	0.191
ALT (U/L)	16 (12.6, 23.1)	35.7 (26, 56)	16.2 (14.4, 21)	0.904	<0.001 ***	<0.001 ***
AST (U/L)	18.8 (16.6, 25.1)	34 (23, 42)	24.8 (19.6, 28.2)	0.034 *	0.003 **	<0.001 ***
GGT (U/L)	18.7 (12.1, 28.6)	94.9 (38, 136)	23.5 (14.4, 27)	0.625	<0.001 ***	<0.001 ***
ALP (U/L)	55.5 (43.8, 71.7)	134 (87, 309)	61.9 (51.8, 75.9)	0.222	<0.001 ***	<0.001 ***
TBA (μmol/L)	3.5 (2.46, 6.2)	11 (8, 16)	3.6 (3.3, 6.85)	0.484	<0.001 ***	<0.001 ***
C3 (g/L)	1.10 (0.92, 1.23)	1.16 (1, 1.36)	1.2 (1.01, 1.37)	0.041 *	0.595	0.080
C4 (g/L)	0.25 (0.18, 0.33)	0.22 (0.15, 0.26)	0.25 (0.19, 0.26)	0.399	0.327	0.072
IgA (g/L)	2.58 (1.91, 3.21)	2.62 (1.75, 3.36)	2.74 (2.12, 4.05)	0.641	0.707	0.904
IgG (g/L)	15.2 (13.7, 16.8)	14.2 (11.6, 16.1)	12.2 (11.2, 13.1)	<0.001 ***	0.013 *	0.031 *
IgM (g/L)	1.21 (0.83, 1.58)	1.57 (0.98, 2.84)	1.29 (0.71, 1.54)	0.625	0.014 *	0.022 *

*p* ^a^-value = pSS vs. HC group; *p* ^b^-value = PBC vs. HC group; *p* ^c^-value = pSS vs. PBC group; WBC: white blood cells; Hb: hemoglobin; PLT: platelet; ESR: erythrocyte sedimentation rate; ALT: alanine aminotransferase; AST: aspartate aminotransferase; GGT: gamma-glutamyl transpeptidase; ALP: alkaline phosphatase; TBA: total bile acid; C3: complement component 3; C4: complement component 4; IgA: immunoglobulin A; IgG: immunoglobulin G; IgM: immunoglobulin M; * *p* < 0.05; ** *p* < 0.01; *** *p* < 0.001.

**Table 3:** Comparison of serological indexes between pSS patients with an active and inactive disease.

	Active (*n* = 12)	Inactive (*n* = 30)	*p*-Value
PLT (10~9/L)	140 ± 52	217 ± 50	<0.001 ***
ESR (mm/1 h)	13.5 (7.75, 38.5)	18 (14, 26)	0.512
AST (U/L)	20.5 (15.8, 25.2)	18.6 (17.3, 24.6)	0.749
C3 (g/L)	0.89 ± 0.32	1.14 ± 0.19	0.002 **
IgG (g/L)	24.5 (17.3, 30.80)	14.45 (13.6, 15.6)	<0.001 ***

** *p* < 0.01; *** *p* < 0.001.

**Table 4:** Comparison of serological indexes between PBC patients with mild and severe cholestasis.

	Mild (*n* = 26)	Severe (*n* = 12)	*p*-Value
ESR (mm/1 h)	13.35 (9, 23)	16 (11.65, 29.5)	0.198
ALT (U/L)	35.2 ± 18.1	52.8 ± 22.1	0.013 *
AST (U/L)	32.3 ± 13.2	44.2 ± 21	0.041 *
GGT (U/L)	52.8 (33, 95.8)	150 (136, 209)	<0.001 **
ALP (U/L)	102 (69, 134)	334.5 (310, 398.5)	<0.001 **
TBA (μmol/L)	9 (7, 12)	16 (15, 25)	<0.001 **
IgG (g/L)	12.9 (11.6, 16.1)	15 (11.07, 16.1)	0.875
IgM (g/L)	1.89 (1.02, 3.53)	1.32 (0.94, 2.25)	0.307

* *p* < 0.05; ** *p* < 0.01.

## Data Availability

The datasets generated and/or analysed during the current study are available in the Figshare repository at https://doi.org/10.6084/m9.figshare.28227677.

## Supplementary Materials

The following supporting information can be downloaded at: https://www.mdpi.com/article/10.3390/microorganisms13122668/s1, Table S1: Analysis of the gut microbiota at the phylum level in PBC and pSS patients and HCs; Table S2: Analysis of the gut microbiota at the genus level in PBC and pSS patients and HC; Table S3: Comparison of the gut microbiota at the genus level between active and inactive pSS patients; Table S4: Comparison of the gut microbiota at the genus level between PBC patients with mild and severe cholestasis.

## Abbreviations

The following abbreviations are used in this manuscript:

PBC	Primary biliary cholangitis
pSS	Primary Sjögren’s syndrome
HC	Healthy control
ALP	Alkaline phosphatase
ULN	Upper limit of normal
ACR	American College of Rheumatology
PCoA	Principal coordinate analysis
SD	Standard deviation
ALT	Alanine aminotransferase
AST	Aspartate aminotransferase
GGT	Gamma-glutamyl transpeptidase
TBA	Total bile acid
IgM	Immunoglobulin M
IgG	Immunoglobulin G
C3	Complement component 3
IgA	Immunoglobulin A
C4	Complement component 4
PLT	Platelets
PCoA	Principle Coordinate Analysis
LEfSe	Linear Discriminant Analysis Effect Size
Hb	Hemoglobin
AIDs	Autoimmune diseases
