# Supplementary material for: Decoding the Gut Microbiome in Primary Sjögren’s Syndrome and Primary Biliary Cholangitis: Shared Dysbiosis, Distinct Patterns, and Associations with Clinical Features
Source: Microorganisms. 2025 Nov 24;13(12):2668. doi: 10.3390/microorganisms13122668 (PMC12734952; doi:10.3390/microorganisms13122668)
Supplement: Supplementary file 1 [file microorganisms-13-02668-s001.zip › microorganisms-3918747-supplementary.pdf]

Table S1. Analysis of the gut microbiota at the phylum level in PBC and pSS patients and HCs.

Phyla	PBC	pSS	HC	P^A-value	P^B-value	P^C-value
Actinobacteria	8.45%	14.31%	3.16%	0.000**	0.039*	0.015*
Proteobacteria	16.36%	8.15%	5.51%	0.101	0.001**	0.025*
Bacteroidetes	16.17%	14.25%	26.77%	0.062	0.378	0.288
Firmicutes	58.45%	62.42%	64.21%	0.639	0.145	0.250

P^A: pSS group vs. HC group, P^B: PBC group vs. HC group, P^C: pSS group vs. PBC group. *P<0.05, **P<0.01, ***P<0.001.

Table S2. Analysis of the gut microbiota at the genus level in PBC and pSS patients and HC.

Genus	pSS	PBC	HC	P^a-value	P^b-value	P^c-value
Bacteroides	11.74%	13.04%	23.32%	<0.001***	<0.001***	0.8342
Faecalibacterium	12.34%	5.01%	9.09%	0.463	0.313	0.004**
Blautia	7.28%	8.76%	8.20%	0.939	0.978	0.789
Bifidobacterium	13.13%	5.08%	2.55%	<0.001***	0.639	0.001**
Shigella	4.43%	9.01%	3.40%	0.926	0.111	0.107
Roseburia	3.70%	4.05%	6.65%	0.531	0.621	0.987
Coprococcus	3.18%	2.04%	3.31%	0.999	0.893	0.870
Gemmiger	3.26%	1.93%	1.51%	0.799	0.987	0.827
[Ruminococcus]	1.19%	2.30%	2.20%	0.965	0.996	0.978
Streptococcus	1.08%	3.25%	1.20%	0.999	0.744	0.604

P^a = relative abundance difference between the pSS group and HC group; P^b = relative abundance difference between the PBC group and HC group; and P^c = comparison of relative abundance of the top 10 gut microbiota between the pSS group and PBC group.

Table S3. Comparison of the gut microbiota at the genus level between active and inactive pSS patients.

Genus	Active	Inactive	P-value
Faecalibacterium	12.02%	11.92%	0.982
Bifidobacterium	9.34%	15.13%	0.401
Bacteroides	11.63%	11.96%	0.954
Blautia	6.94%	7.59%	0.839
Roseburia	7.25%	2.24%	0.002**
Shigella	3.72%	4.87%	0.753
Gemmiger	4.61%	2.68%	0.273
Coprococcus	2.50%	3.53%	0.504
Ruminococcaceae_Ruminococcus	3.01%	1.87%	0.332
[Ruminococcus]	1.28%	1.17%	0.872

Table S4. Comparison of the gut microbiota at the genus level between PBC patients with mild and severe cholestasis.

Genus	Mild	Severe	*P*-value
Bacteroides	11.20%	17.02%	0.410
Shigella	5.08%	17.54%	0.205
Blautia	11.11%	3.68%	0.003**
Bifidobacterium	3.59%	8.30%	0.351
Faecalibacterium	4.64%	5.82%	0.652
Roseburia	4.51%	3.04%	0.462
Lactobacillus	3.92%	1.63%	0.549
Streptococcus	4.42%	0.74%	0.251
Psychrobacter	5.12%	0.00%	0.164
Collinsella	3.39%	0.72%	0.475
